# High-performance PCB-based capillary pumps for affordable point-of-care diagnostics

**DOI:** 10.1007/s10404-017-1935-2

**Published:** 2017-05-24

**Authors:** Nikolaos Vasilakis, Konstantinos I. Papadimitriou, Hywel Morgan, Themistoklis Prodromakis

**Affiliations:** Electronics and Computer Science Department, University of Southampton, Southampton, SO17 1BJ Hampshire United Kingdom

**Keywords:** Capillary pumps, Lab-on-PCB, Microfluidics, Micropillars, Point-of-care diagnostics, Resistance flow

## Abstract

Capillary pumps are integral components of passive microfluidic devices. They can displace precise volumes of liquid, avoiding the need for external active components, providing a solution for sample preparation modules in Point-of-Care (PoC) diagnostic platforms. In this work, we describe a variety of high-performance capillary pump designs, suitable for the Lab-on-Printed-Circuit-Board technology (LoPCB). Pumps are fabricated entirely on Printed Circuit Board (PCB) substrates via commercially available manufacturing processes. We demonstrate the concept of LoPCB technology and detail the fabrication method of different architectures of PCB-based capillary pumps. The capillary pumps are combined with microfluidic channels of various hydraulic resistances and characterised experimentally for different micropillar shapes and minimum feature size. Their performance in terms of flow rate is reported. Due to the superhydrophilic properties of oxygen plasma treated FR-4 PCB substrate, the capillary pump flow rates are much higher (138 μL/min, for devices comprising micropillar arrays without preceding microchannel) than comparable devices based on glass, silicon or polymers. Finally, we comment on the technology’s prospects, such as incorporating more complicated microfluidic networks that can be tailored for assays.

**Electronic supplementary material:**

The online version of this article (doi:10.1007/s10404-017-1935-2) contains supplementary material, which is available to authorized users.

## Introduction

A major driver behind the development of microfluidic devices is to perform complex biological and chemical processes and measurements rapidly and efficiently with miniaturised devices (Yager et al. 2006). Over the last 15 years, microfluidic devices based on polymers and paper materials have been developed, setting new standards for affordable, easily disposable and versatile inexpensive medical diagnostic platforms (Coltro et al. 2014; Gervais et al. 2011; Yetisen et al. 2013; Zanoli and Spoto 2013). Microfluidic PoC devices have been developed (Adkins et al. 2015; Vashist et al. 2015; Xia et al. 2016), demonstrating in many cases a *sample*-*to*-*answer* capability (Jung et al. 2015). However, developing PoC tests that perform multiple functions in a monolithic manner, while meeting the “ASSURED” criteria (affordable, sensitive, specific, user friendly, robust and rapid, equipment-free and deliverable to end-users) (Urdea et al. 2006) remains a challenge (Jung et al. 2015; Su et al. 2015). The key for a successful PoC device is integration of microfluidics, detection and signal processing in one platform. To address this, an alternative microfluidic system based entirely on PCB manufacturing technology is demonstrated in this work: the LoPCB. The key aspect of this technology is the integration of microfluidic components with electronics, heaters, electrodes and biosensors into a monolithic device. The wetting properties of the core material (flame retardant grade 4, FR-4) can be altered with O_2_ plasma treatment thus delivering channels with different capillary flow characteristics. In this paper, a key component of this technology is described which is a capillary pump that exploits the superhydrophilic properties of the O_2_ plasma treated FR-4. The micropump is based on micropillar arrays and provides accurate and tunable control of fluid flow across a wide range of flow rates. The fabrication process is based on the well-established PCB manufacturing technology. The capillary pumps are fabricated and tested, and their potential for integration into PoC diagnostic platforms is discussed.

The mixed circuit board (MCB) concept was initially proposed and demonstrated by Lammerink et al. (1996) and further developed by Merkel et al. (1999). Their work exploits a PCB manufacturing technology to fabricate microfluidic platforms combined with electronic components. Their prototype devices comprised standard PCB features such as copper tracks, combined with electronic assemblies, functional microfluidic components and sensing electrodes, all fully integrated in a monolithic manner, i.e. a fully functional Lab-on-PCB (LoPCB) technology. The latter benefits from the high degree of electronics integration, high precision and the accumulated experience and skills of a mature industrial manufacturing process (Bachman and Li 2012; Wu et al. 2011). The LoPCB technology provides an analytical platform where the microfluidic components for sample preparation and reagent manipulation can be integrated with electrochemical sensors and circuits. Hence, the footprint required for the whole measuring set-up is smaller, since there is no need for separate electronics and assay platforms. In contrast to bulky high-sensitivity spectrometric apparatus, LoPCB technology can offer direct electrochemical sensing in mm space, using mature electronic techniques (Hartman et al. 2013). Moreover, combining both biochemistry and electronic biosensing on the same platform reduces noise interference due to connectivity issues and increases the signal-to-noise ratio (SNR).

**Fig. 1:**
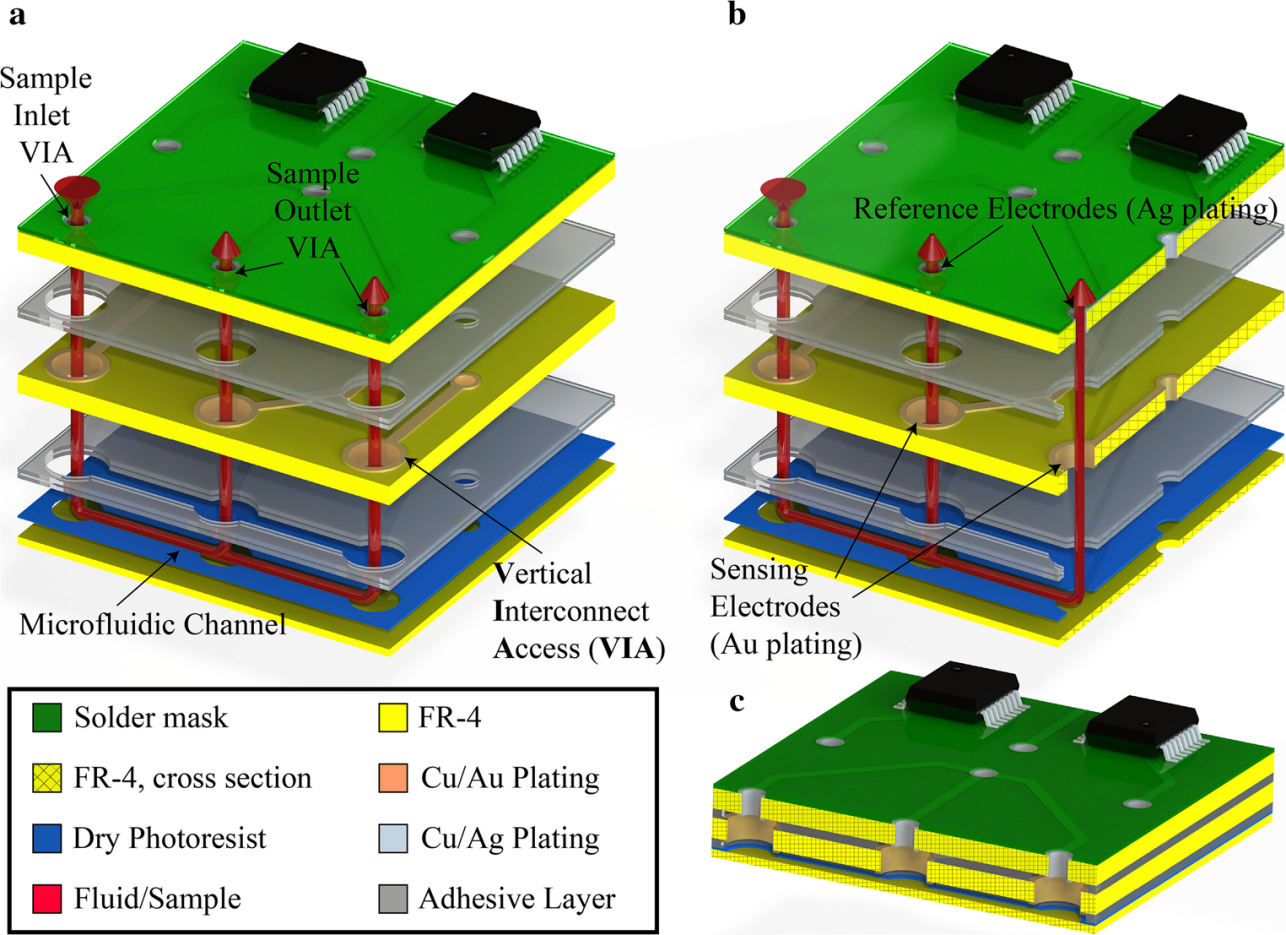
LoPCB technology. **a** Exploded view, **b** exploded view–cross-sectional view, **c** cross-sectional view along the microfluidic channel

The microfluidic components for sample and reagent manipulation can be integrated with gold (Au)- or silver (Ag)-plated sensing pads, enabling electrochemical detection of molecular events (Papadimitriou 2016a, b; Vasilakis et al. 2016b). A generalised architecture of the LoPCB technology is shown in Fig. 1, which illustrates a standard “3-layer” LoPCB architecture, with integrated microfluidics, biosensors and electronics. The inner bottom layer is a photo-lithographically patterned dry photoresist laminated on an FR-4 substrate that can be modified as required to form the integrated fluidic control and pump structure. PCB-based devices such as micromixers (Aracil et al. 2015), nucleic acid amplification chips (Moschou et al. 2014) and chemical sensors (Prodromakis et al. 2011) have been demonstrated in the literature. The LoPCB approach thus satisfies most of the criteria for autonomous monolithic PoC platforms, as discussed earlier. The intrinsic hydrophobic properties of PCB substrates, e.g. flame-retardant grade 4 (FR-4), will require  external actuation, e.g. a syringe pump, for pumping fluidic samples. This leads to tubing connection issues, loss of analytes in the dead volume of the tubing and most important decreased compactness and portability, a drawback severely affecting the adaptation of LoPCB technology for chemical and bio-analytical platforms. Owing to these issues, a LoPCB device suitable for a PoC has not been implemented to date. All the published devices based on PCB technology use syringe pumps to move samples and reagents within the microfluidic channels placing limits on their applicability to PoC. Thus, LoPCB devices with in-built fluid control based on capillary forces would deliver the equipment-free requirement included in the ASSURED criteria eliminating both fluid interfacing and external power consumption. To address this, we developed a capillary pumping system that is easily integrated into a LoPCB system to facilitate the accurate and effective manipulation of fluids.

Capillary pumping is an elegant and useful method of moving liquids in passive microfluidics (Zimmermann et al. 2007). The flow rate in these devices is design-dependent and controlled by the surface tension of the liquids, microchannel wall wettability and geometry (i.e. width, height and length) (Delamarche et al. 1998). Several studies have exploited capillary forces for liquid transportation within a microfluidic network. Juncker et al. (2002) reported an autonomous microfluidic capillary system transporting aliquots of different liquids from the inlets through a reaction chamber into the capillary pumping component comprising three microchannels. The performance of this technology was enhanced by Zimmermann et al. (2007), who studied the effect of various capillary pumping structures, while more recently Madadi et al. (2014) optimised the shape of micropillars and studied the effect of the geometry of the pillar array.

Inspired by the previous studies on capillary driven pumps, we developed a pumping technique to control fluid movement in a PCB Lab-on-Chip. In our previous work we demonstrated that oxygen plasma (O_2_ plasma) treatment of FR-4 surfaces rendered the surface long-lastingly hydrophilic, with devices retaining superhydrophilicity for 13 days, and remaining weakly hydrophilic for another 13 days (Vasilakis et al. 2016a). Plasma reactors are used as etching machines in standard PCB manufacturing processes and provide an environmental-friendly way of cleaning and etching PCBs (Schmidt et al. 1998). In this paper, we combine the concept of micropillar-based capillary microfluidic structures with plasma processing to create a fully functional PCB-based capillary pump. This is the first demonstration of micropillar array capillary pumps combined with microfluidic channels to control the flow rate for LoPCB devices fabricated with standard PCB manufacturing techniques. Two different pump architectures were designed and fabricated, each with three different minimum feature sizes (MFS). Experimental data demonstrate the performance of the various pumps.

## PCB-based micropillar designs

Capillary pumps comprise either many parallel microchannels with a common inlet or micropillar array structures to provide the desirable capillary pressure. Micropillar structures are of greater interest, mainly due to the increased surface-to-volume ratio enhancing the capillary flow and their ability to minimise entrapped air (Zimmermann et al. 2007). Micropillar structures can be very versatile as well. By modifying a range of geometrical parameters, significant differences in the performance and accuracy of the pump can be achieved. The micropillar shape can be optimised to minimise flow resistance, while the liquid filling front advances in the capillary pump. Moreover, the desired flow rate through the device can be tailored by introducing a microfluidic flow resistance at appropriate points. The flow properties of autonomous capillary systems were investigated by Zimmerman et al. (2007) for various micropillar shapes.

**Fig. 2:**
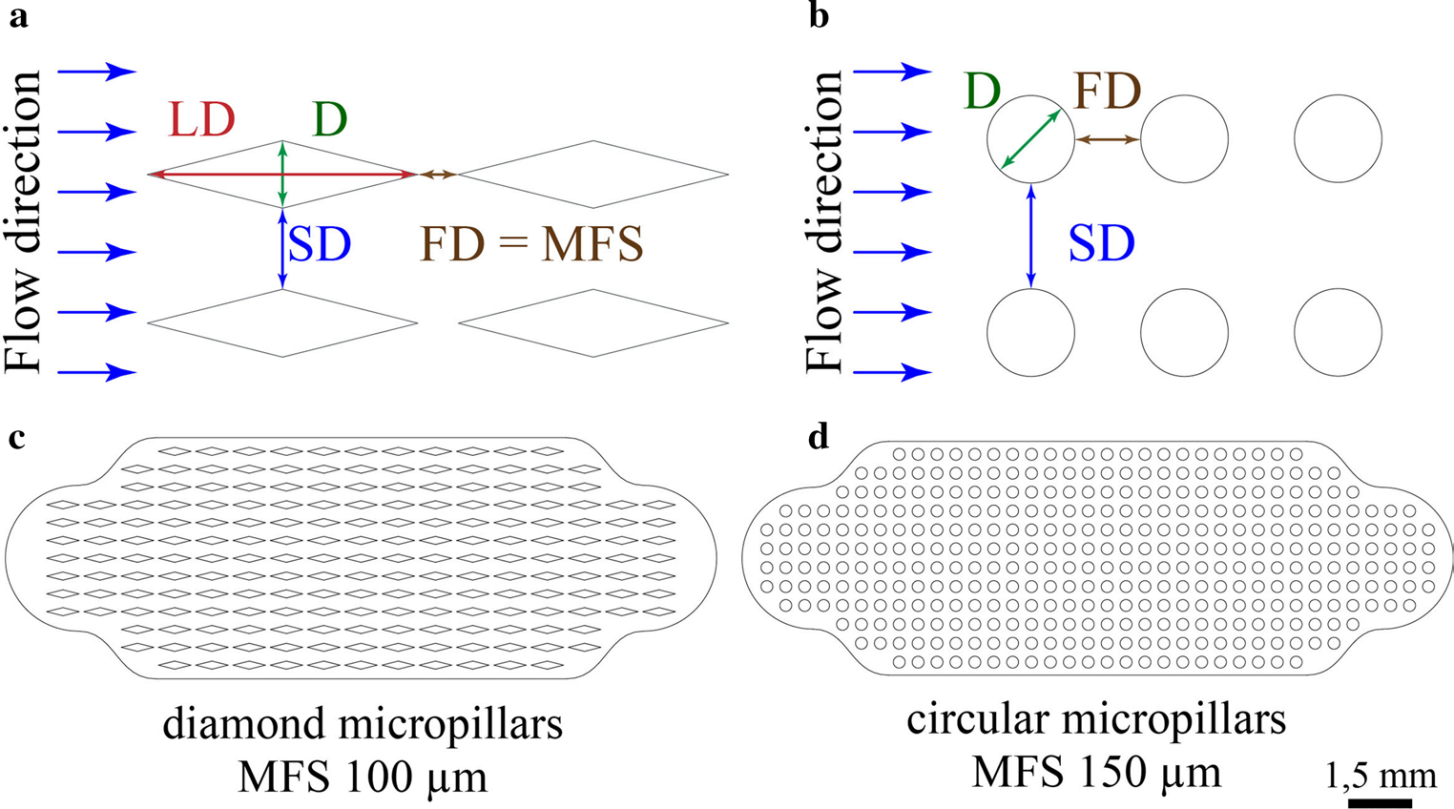
Micropillar shape and array topology parameters for: **a** diamond micropillars, **b** circular micropillars. PCB microfluidic capillary pump designs: **c** diamond pillar design with MFS 100 μm, **d** circular pillar design with MFS 150 μm

In the work of Madadi et al. (2014), an extensive investigation of both the micropillar geometry and array topology on the hydraulic resistance was presented. The authors examined a large variety of micropillar shapes and offered a detailed comparison between these, describing optimal architectures to ensure minimum flow resistances. Figure 2 illustrates two of these proposed architectures, i.e. a diamond (Fig. 2a) and a circular micropillar shape (Fig. 2b), together with geometrical parameters, e.g. the long diagonal (LD) and the short diagonal (D). In this work, we studied the performance of both circular and diamond-shaped micropillar arrays. Circular micropillars are easier to fabricate, while the diamond-shaped micropillar arrays of Madadi et al. are reported to achieve higher flow rates. Adopting the nomenclatures of Madadi et al. we define the micropillar array by the flow directional distance FD and side distance SD between the micropillars, while the minimum feature size (MFS) of the structure is stipulated by FD. Figure 2c and d shows examples of the designed and fabricated capillary pumps. A total of six different architectures were selected for analysis consisting of three variations of the diamond and circular structures, each defined by a different MFS, namely 50, 100 and 150 μm. The PCB-based capillary pumps were also combined with three variations of 250-μm-wide serpentine microchannels with 2, 10 and 20 turns, corresponding to a total length of 14.71, 72.00 and 145.55 mm, respectively. Thus, a total of 24 different combinations were fabricated and characterised. Table 1 provides a summary of all design parameters and the resulting pump capacities.

**Table 1:** Micropillar capillary pump design parameters

Micropillars shape	MFS (μm)	LD (μm)	D (μm)	SD (μm)	FD (μm)	Pump volume (μL)
Circular	150	–	250.00	150.00	150.00	1.38
Circular	100	–	166.67	100.00	100.00	1.79
Circular	50	–	83.33	50.00	50.00	1.80
Diamond	150	1000.00	250.00	300.00	150.00	2.09
Diamond	100	666.67	166.67	200.00	100.00	2.04
Diamond	50	333.33	83.33	100.00	50.00	2.10

Equations (1) and (2) define the relation between the design parameters (MFS, FD, D, SD and LD) of the diamond and circular architectures, respectively, derived from the experimental results in micropillar capillary pump flow rate performance of Madadi et al. (2014).

1 $${\text{MFS}} = {\text{FD}} = \frac{3}{5}{\text{D}} = \frac{\text{SD}}{2} = \frac{3}{20}{\text{LD}}$$

2 $${\text{MFS}} = {\text{FD}} = \frac{3}{5}{\text{D}} = {\text{SD}}$$

## Materials and methods

**Fig. 3:**
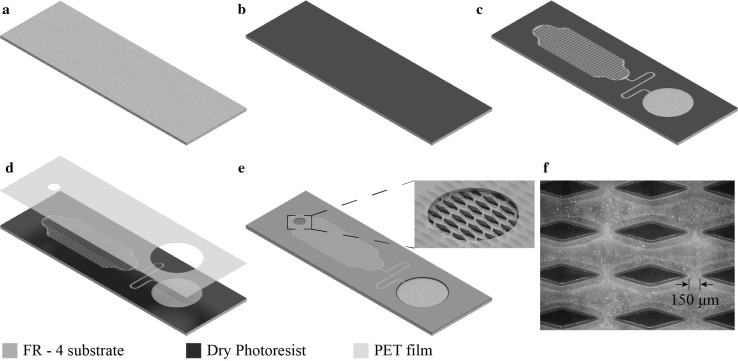
Sample fabrication steps. **a** FR-4 laminate cleaning, **b** dry photoresist lamination, **c** exposure, development and O_2_ plasma radiation, **d** PET film laser micromachined, **e** micromachined PET film lamination, **f** microscope image of the diamond micropillars

The fabrication process is described in Fig. 3 and is similar to the standard industrial PCB manufacturing processes. As a first step, a 400-μm-thick FR-4 laminate was cleaned using acetone and isopropanol (IPA) (Fig. 3a). Subsequently, DuPont™ Pyralux^®^ PC1000 negative dry photoresist, 50 μm thickness, was laminated on top of the FR-4 laminate, using a pouch laminator (Fig. 3b). The sample was exposed through a mask using hard contact (using an EVG 620 mask aligner) and developed in a mild caustic solution (sodium carbonate 1% w/w), following standard lithographic processes. The developed structures (Fig. 3c) were post-baked at 160 °C for 2 h. After post-baking, the samples were irradiated with O_2_ plasma to render the FR-4 substrate superhydrophilic (contact angle ≈ 0°). The O_2_ plasma treatment conditions were identical to those used previously by our group (Vasilakis et al. 2016a). Following O_2_ plasma treatment, a polyethylene terephthalate sealing film (50 μm thickness, PET, VWR^®^ Polyester Sealing Films for ELISA) was machined using a CO_2_ laser cutter (Epilog Mini 24 Legend Laser System, USA) to define sample inlet and outlet vent holes (Fig. 3d). The machined PET film was laminated on top of the samples with a pouch laminator (Fig. 3e). The resulting internal channel height was around 40 μm, based on standard stylus profilometer measurements. Finally, Fig. 3f illustrates a microscope image of the fabricated diamond micropillars of the PCB-based capillary pump (see Fig. 2c).

A “first-order” analysis of the rectangular microchannel, i.e. the first segment of the fabricated PCB-based capillary pumps (see Fig. 2), can be performed using the following Eqs. (3)–(6). The capillary pressure *P* formed in a rectangular microchannel driving the liquid–air meniscus through the channel is defined as:

3 $$P = - \gamma \left( {\frac{{\cos \theta_{\text{B}} + \cos \theta_{\text{T}} }}{\text{ch}} + \frac{{2 \cdot \cos \theta_{\text{W}} }}{\text{cw}}} \right)$$

where *γ* is the surface tension of the liquid in N/m, cw and ch are the microchannel width and height in metres, and *θ*_B_, *θ*_T_, *θ*_W_ are the contact angles of the liquid on the bottom, top and side walls, respectively. The resulting flow rate *Q* of the liquid, when filling a microchannel by capillary pressure *P*, is:

4 $$Q = \frac{{\Delta P}}{{\mu \cdot L \cdot R_{\text{cs}} }}$$

where *μ* is the dynamic viscosity of the liquid in Pa·s, *L* the length of the filled microchannel (in m), and *R*_cs_ is the cross-sectional hydraulic resistance parameter for rectangular microchannels in m^−4^. The parameter *R*_cs_ of Eq. (4) can be calculated from Eq. (5) (Spurk and Aksel 2006):

5 $$R_{\text{cs}} = \left[ {\frac{{A \cdot {\text{ch}}^{2} }}{4} \cdot \left( {\frac{1}{3} - \frac{{{\text{ch}} \cdot 64}}{{{\text{cw}} \cdot \pi^{5} }}\sum\limits_{n = 1}^{\infty } {\frac{{\tanh \left( {m \cdot \frac{\text{cw}}{2}} \right)}}{{\left( {2n - 1} \right)^{5} }}} } \right)} \right]^{ - 1}$$

with *A* the cross-sectional area of the microchannel in m^2^ and ch and cw defined above. For the Fourier series in Eq. (5), only the first six terms were used to calculate *R*_cs_, producing a negligible error of ~10^−6^. Finally, the parameter *m* (m^−1^) in Eq. (5) is described as follows:

6 $$m = \frac{\pi }{\text{ch}}\left( {2n - 1} \right)$$

To investigate the time-dependent behaviour of the liquid within the microfluidic channel, additional mathematical analysis was conducted. More specifically, Eq. (4) can be rewritten as a function of the meniscus position in the microchannel if *L* is substituted by *x*, the distance of the advancing meniscus in the microchannel form the inlet (in metres):

7 $$Q_{(x)} = \frac{B}{x} = A \cdot \overline{{u_{(x)} }} ,\quad {\text{where}}\;B = \frac{{\Delta P}}{{\mu \cdot R_{\text{cs}} }}$$

With the mean flow velocity $\overline{{u_{(x)} }}$ described as a function of the time derivative of the displacement:

8 $$\overline{{u_{{\left( {x_{(t)} } \right)}} }} = \frac{\text{d}x_{(t)}}{\text{d}t} = \frac{B}{A} \cdot \frac{1}{{x_{(t)} }}$$

we end up with an ODE, with a compact form:

9 $$\frac{\text{d}x_{\left( t \right)}}{\text{d}t}- \frac{B}{A} \cdot \frac{1}{{x_{\left( t \right)} }} = 0$$

A solution to the ODE of Eq. (9), defining the time-dependent behaviour of the resulting flow in the microfluidic channel, is:

10 $$Q_{\left( t \right)} = \frac{B}{{\sqrt {\frac{2B}{A} \cdot t} }} = \sqrt {\frac{A \cdot B}{2 \cdot t}}$$

Equation (10) is useful in providing an analytical description of the flow rate as a function of time within a rectangular microfluidic channel. As shown in the following section, Eqs. (3)–(10) were used to simulate the flow rate of the fluid within the microchannel of the capillary pumps.

## Experimental results and discussion

**Fig. 4:**
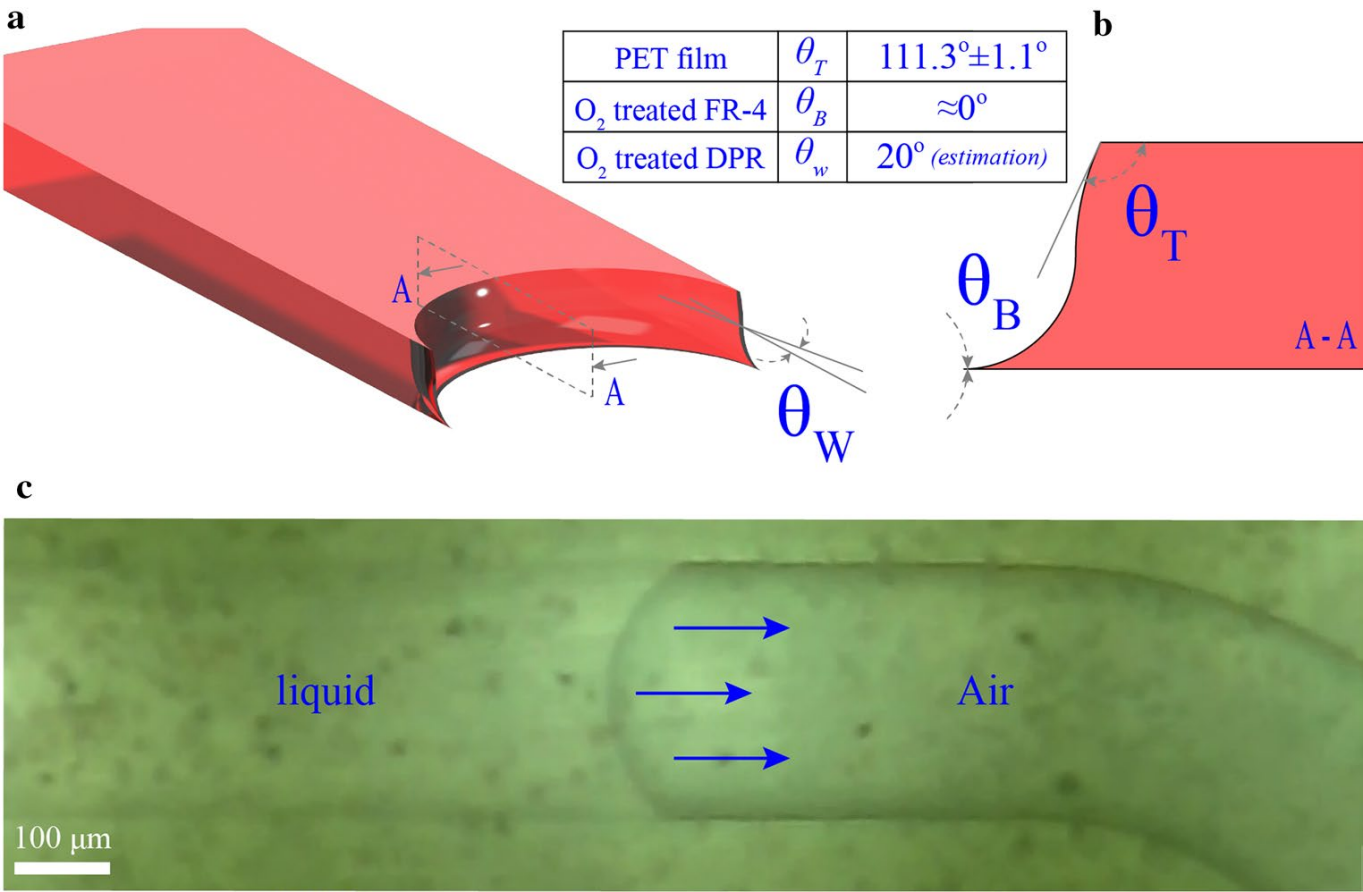
Liquid meniscus advancing in the hydrophilic microchannel: **a** 3D model of the formed meniscus, **b** A–A sectional view of the meniscus along the microchannel central axis, **c** still image of the actual meniscus advancing in the microchannel

Red dye (Direct Red 23, Sigma-Aldrich^®^) dissolved in deionized (DI) water was used as sample solution for characterisation. Initially, a 6 μL sample droplet was deposited in the 6-mm-wide device inlet. The filling of the device was recorded with a full HD (1080p) digital microscope camera at a 50 Hz frame rate. As a first characterisation step, the static contact angles (CA) of sessile deionised (DI) water droplets on the PET film were measured with a Drop Shape Analysis System (DSA30 Kruss Co., Germany), see Vasilakis et al. (2016a). A 3-μL DI water droplet was placed on the adhesive surface of the PET film. Each measurement was repeated three times, giving a CA of 111.3° ± 1.1°, verifying hydrophobic behaviour of the PET film used to seal the devices. The O_2_ plasma treated FR-4 surface has superhydrophilic properties, and hence the value of the CA can be approximated to ≈0° (Vasilakis et al. 2016a). Figure 4 illustrates a mathematical representation (Fig. 4a, b), and an experimental image of the shape of the meniscus formed as the liquid advances in the microchannel (Fig. 4c). More specifically, in Fig. 4a, the shape of the advancing meniscus in the microchannel is demonstrated geometrically, while in Fig. 4b, a sectional view along the microchannel central axis is shown. Both images were generated using the experimentally measured CAs of the selected materials (O_2_ plasma treated FR-4 and PET film). The CA *θ*_T_ on the top wall is assumed based on the PET hydrophobic behaviour (*θ*_T_ = 111.3° ± 1.1°). The meniscus angle formed on the bottom O_2_-treated FR-4 surface *θ*_B_ is considered ≈0°, while the CA on the microchannel dry photoresist (DPR) sidewalls *θ*_W_ was assumed to be 20°, determined from the meniscus images (please see Fig. 4c) and the reported effect of O_2_ treatment on DPR (Kalkandjiev et al. 2010). Figure 4c verifies the expected fluid formation showing an image of the actual liquid–air meniscus advancing in the microchannel.

**Fig. 5:**
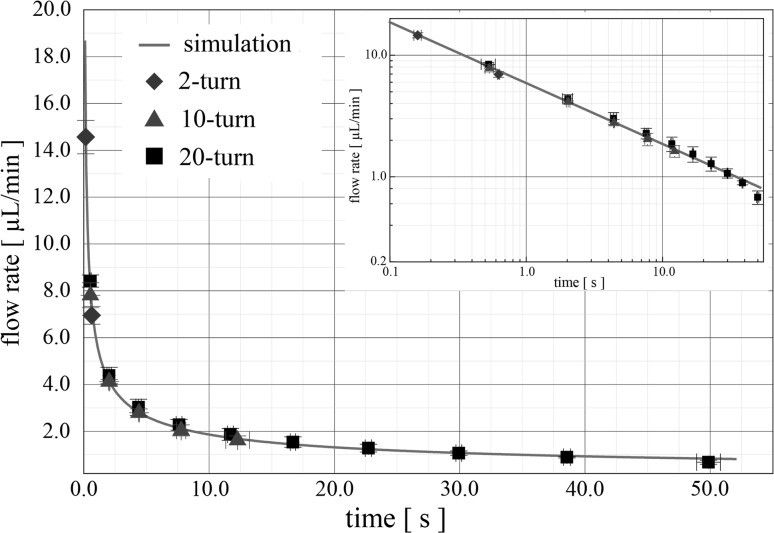
Comparison between the experimental average flow rates and the theoretical flow rate, while the liquid–air meniscus advances in the 2-turn, 10-turn and 20-turn serpentine microchannel

**Fig. 6:**
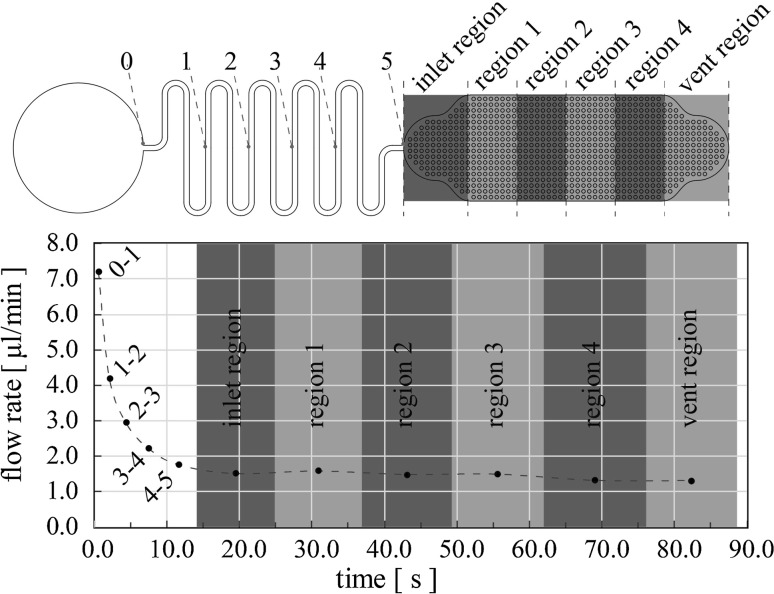
Flow rate experimental characterisation. The device comprises a 10-turn serpentine microchannel divided in five sections. The capillary pump is divided in six regions. The points in the graph are the measured average flow rates, while the liquid front was advancing through the device

In order to validate Eq. (10) describing the liquid meniscus advancing in the microchannel as a function of time, the videos of microchannel filling for different devices were analysed. In particular, the 2-turn microchannel was divided in two equal parts, while the 10- (see Fig. 6) and 20-turn microchannels were divided into 5 and 10 parts, respectively. The number of video frames for the meniscus to reach the particular positions separating the different parts (as illustrated in Fig. 6, points 0–5 divide the microchannel of the 10-turn device into six regions) along the microchannel was counted. Subsequently, the average flow rate for the region to be filled was calculated. In total, nine videos of various devices (three of each design) were processed and the data statistically analysed. Figure 5 summarises the average flow rates as the sample filled the microchannel. The simulation in Fig. 5 demonstrates the ideal flow rate of the liquid within the microfluidic channel as time evolves according to Eq. (10), where the points in the figure show the experimental results from devices comprising 2-, 10- and 20-turn serpentine microchannels. Error bars are standard deviations.

Good agreement was observed between theory and experimental results for all fabricated devices. The theoretical value derived from Eq. (4) assuming *θ*_B_, *θ*_T_ and *θ*_W_ = 0°, 112.4° and 20°, respectively, with *L* = 72 mm (at the end of the 10-turn microfluidic channel, Fig. 6) was found equal to 1.61 μL/min, while the average experimentally measured flow rate for region 4–5 (see Fig. 6) is 1.63 ± 0.18 μL/min, verifying a good match between theory (Eq. 4) and device. Finally, the inset in Fig. 5 demonstrates the same data plotted on a log–log scale, to illustrate the power law dependence in the first seconds of flow.

Figure 6 shows data for the whole device (microchannel and capillary pump). The microchannel was divided into five sections as described previously, while the capillary pump was divided into six regions. For the capillary pump, the first region (inlet region) is the transition zone between the microfluidic inlet and the main uniform capillary pumping zone (region 1–4), while the last region (vent) comprises the air vent of the device. Regions 1 and 4 are of the same liquid capacity. The same applies between regions 2 and 3. However, the volume percentile difference between regions 1 and 2 (or 3 and 4) is below 0.5% for MFS ≤ 100 μm and below 4.0% for MFS = 150 μm.

**Fig. 7:**
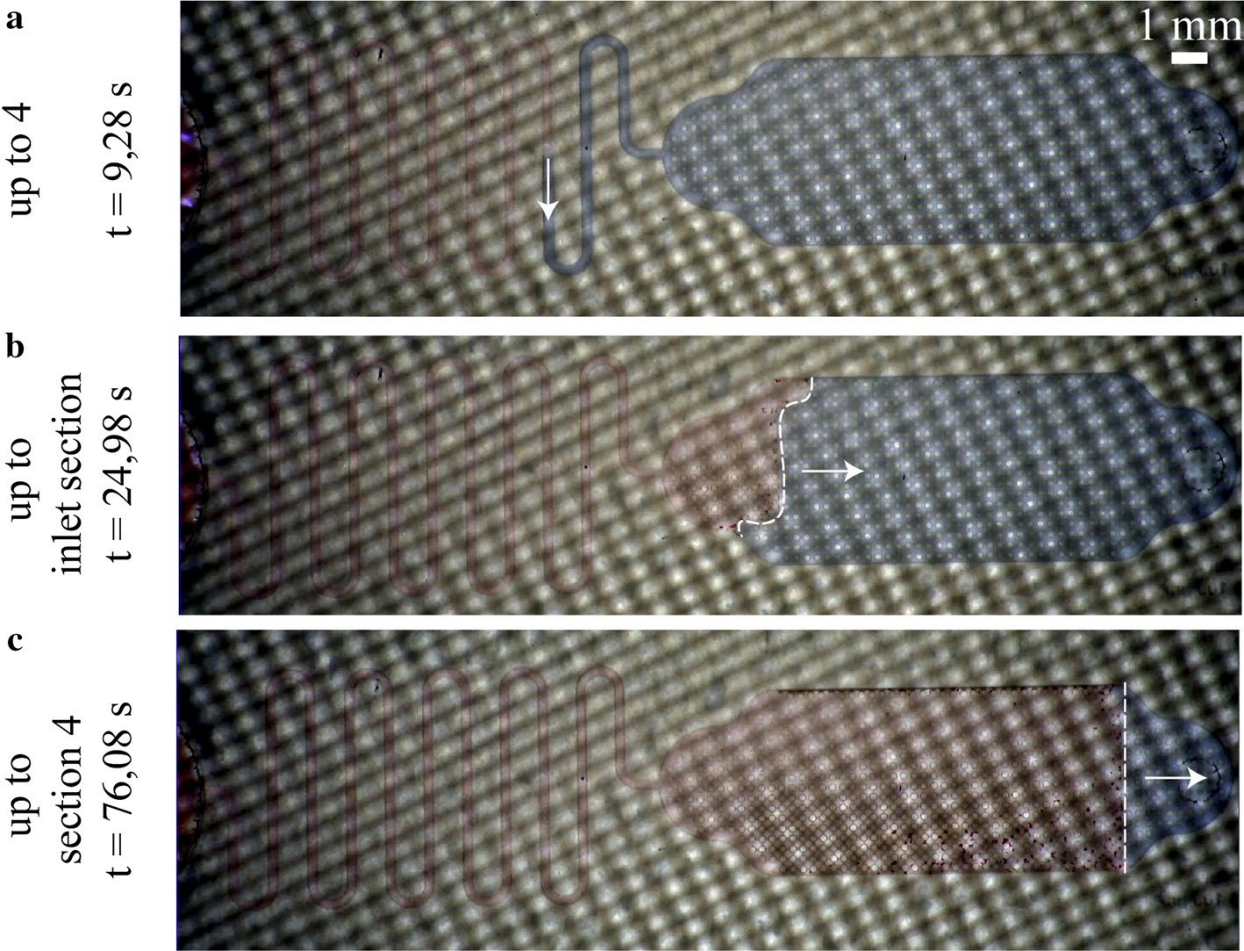
Images taken from the flow rate from videos: **a** sample filling the microchannel. Filling front advancing in (**b**) region 1 (**c**) vent region

The experimentally measured average flow rate for the microfluidic channel is described in Fig. 6, while for every capillary pump zone the experimentally determined average flow rate was calculated from the recorded data. Three example images are shown in Fig. 7. These three images show the propagation of the sample from the microfluidic channel (Fig. 7a), into the inlet region of the capillary pump (Fig. 7b) and then travelling towards the vent (Fig. 7c). Note that the sample in Fig. 7b has just entered region 1, while in Fig. 7c it just entered the vent region. (Videos of the results can be found in ESI).

The mechanism governing filling of capillary pumps is well understood. The microfluidic channel upstream of the capillary pump acts as a constant hydraulic resistance once it is completely filled and the liquid front advances towards the capillary pump. Therefore, devices comprising only capillary pumps tend to exhibit very high flow rates. This is why in our case, the diamond micropillar capillary pump with MFS 100 μm without a microchannel (as shown in Fig. 2c) has an average flow rate of 137.51 μL/min in regions 1 to 4, with the average flow rates filling regions 1 and 4 are ~220 and ~92 μL/min, respectively. The variation in flow rate between regions 1 and 4 is expected, since the flow rate tends to decrease as the filling front advances. This can be attributed to the absence of a microchannel with significantly higher hydraulic resistance compared to the micropillar hydraulic resistance. Additionally, the designs were not long enough to achieve equilibrium between the micropillar hydraulic resistance and the capillary pressure and therefore the flow rate has a large difference between minimum and maximum values. However, the overall performance, capacity and footprint (around 14.6 mm × 5.0 mm) of these devices, makes them an excellent solution for single-step LoPCB analytical platforms, once electrodes are integrated into them. It is worth highlighting that our designs have around 56 times higher average flow rates compared to the capillary pumps reported by Madadi et al. (2014). This difference in flow rate performance is attributed to both the 6.7 times higher micropillar height (our devices use 40-μm-height micropillars instead of 6 μm) reducing the micropillar array hydraulic resistance and the superhydrophilic properties of O_2_-treated FR-4 substrate, which increases the capillary forces.

**Fig. 8:**
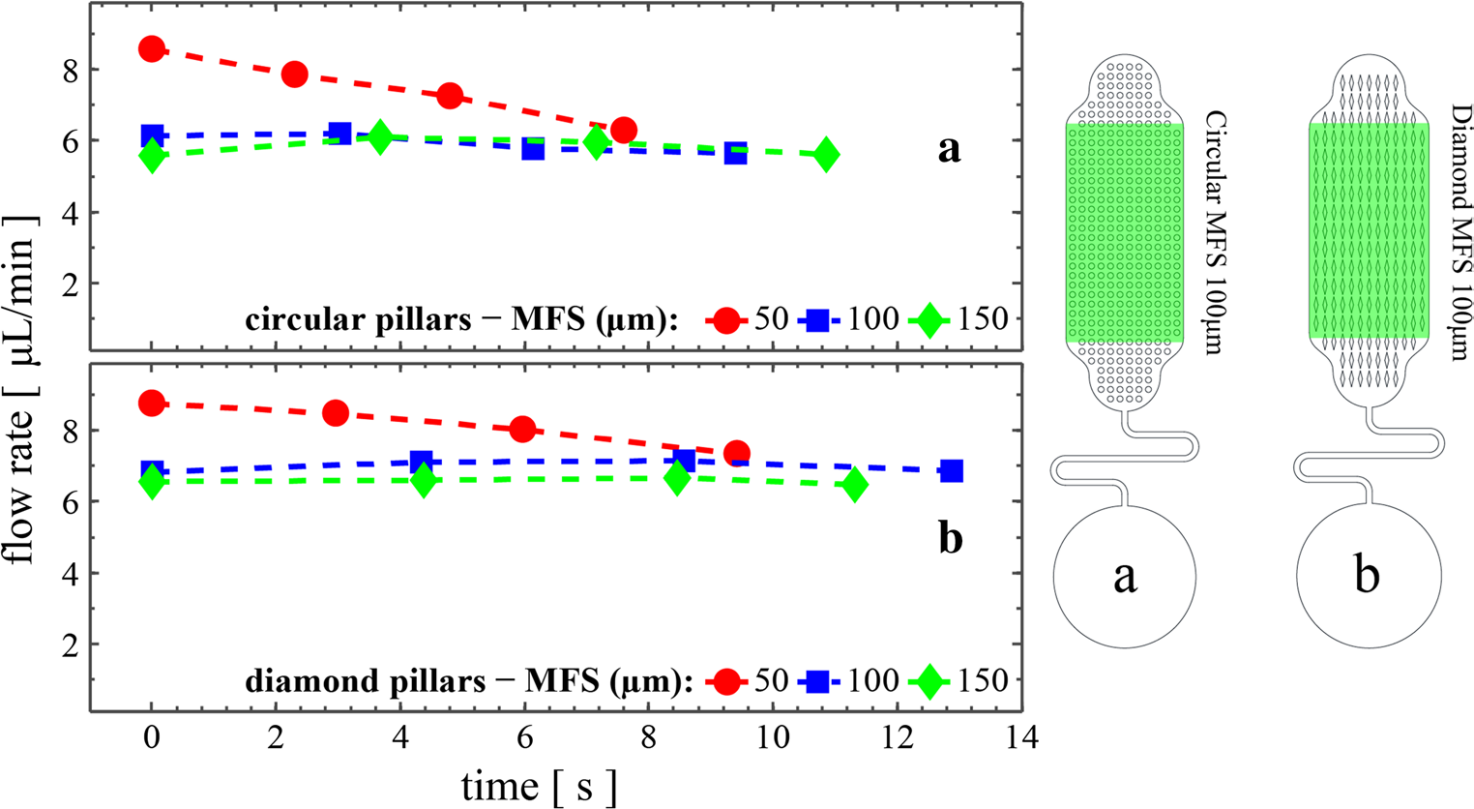
Average flow rates filling regions 1 to 4 of devices comprising capillary pumps having various MFS and 2-turn microchannel: **a** circular micropillars, **b** diamond micropillars

**Fig. 9:**
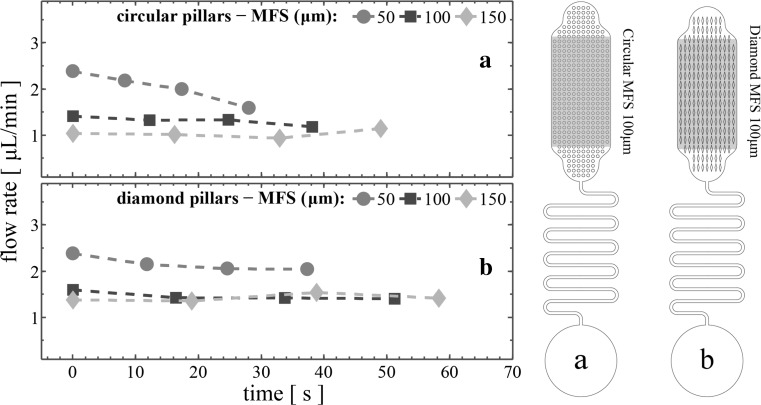
Average flow rates filling regions 1 to 4 of devices comprising capillary pumps having various MFS and 10-turn microchannel: **a** circular micropillars, **b** diamond micropillars

For the other capillary pump architectures, the average flow rate of the sample during propagation through the inlet as well as the vent regions is expected to differ from the average flow rate through regions 1 to 4. This can be explained by the fact that the inlet region comprises micropillar arrays that serve as transition zones between the microchannel and the main pumping regions (1 to 4). The number of micropillar rows in both inlet and vent region differs from the one in regions 1 to 4 where the number of rows is constant throughout. Additionally, the distance between the pillars and the curved pump sidewalls varies in these two regions, affecting the flow rate. Therefore, the performance of the devices was experimentally investigated only when the filling front wetted regions 1 to 4. Figure 8 illustrates the average flow rates of devices combining 2-turn serpentine microchannels with circular and diamond micropillar architectures (Fig. 8a, b, respectively), while Fig. 9 is for the 10-turn serpentine microchannels together with circular and diamond micropillars (Fig. 9a, b, respectively).

As expected, smaller MFS results in higher flow rates. Note that the capacity of the capillary pumps made with diamond micropillars is higher compared to the circular shaped micropillars (see Table 1). For the 2-turn microchannel structures in Fig. 8, the maximum average flow rate (8.11 μL/min ± 9%) was achieved using the diamond-shaped micropillars of 50-μm MFS, despite the fact that these devices exhibit non-constant flow rate (the plus-minus percentile variation is calculated as the ratio of the absolute difference between the maximum and minimum observed flow rates, and the overall average flow rate through regions 1 to 4). More specifically, the average flow rate through region 1 gradually decreased as the filling front advances in the capillary pump. Such behaviour is mainly due to the balance between the capillary pressure formed in the capillary pump and the hydraulic resistance of the microfluidic channel. Thus, devices with longer microchannels such as those in Fig. 9b have a lower flow rate (2.15 μL/min ± 8%), but with less variation. Finally, the diamond micropillars produce higher flow rates compared to the circular pillars, in agreement with Madadi et al. (2014). The 100- and 150-μm MFS devices shown in Figs. 8 and 9 demonstrate stable average flow rates with low variation. In particular, diamond capillary pumps with 100-μm MFS had average flow rates of 7.00 μL/min ± 2% when combined with the 2-turn serpentine microchannel (14.7 mm long).

**Fig. 10:**
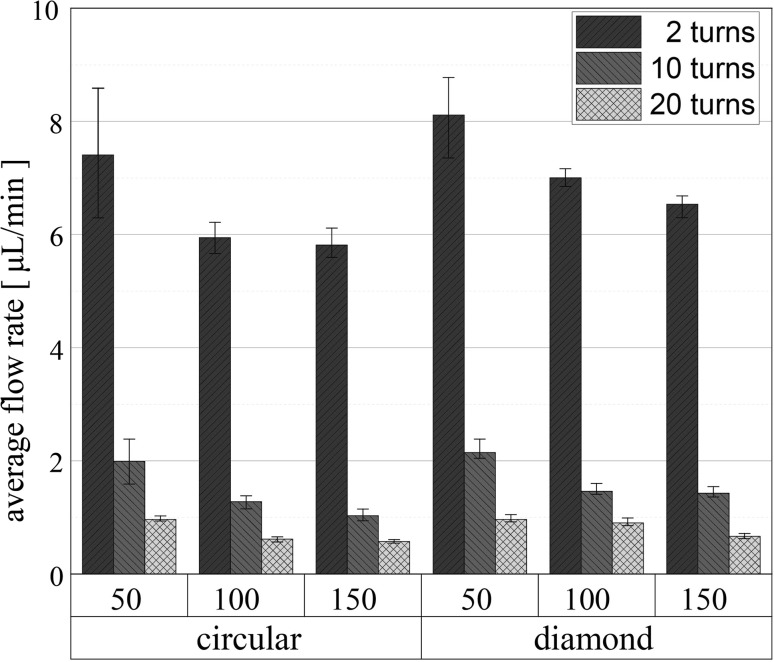
Average flow rates while filling front advances in regions 1 to 4 of devices with circular and diamond micropillars, and 2-, 10- and 20-turn microchannels

Figure 10 summarises the measured average flow rates of all the devices with serpentine microchannels. The error bars represent the minimum and maximum observed average flow rates. The devices comprising 20-turn microchannels give the lowest variation in flow rate for either the MFS or micropillar shape. Compared to Zimmerman et al. (2007), we achieved more than five times higher flow rate with less than three times the microchannel hydraulic resistance. More specifically, our 150-μm MFS diamond-shaped micropillar devices combined with the 20-turn microchannel exhibit an average flow rate of 0.66 μL/min, while Zimmerman’s “symmetric-line”-type devices with characteristic length 150 μm [the 150_R1 design (Zimmermann et al. 2007)] have a flow rate of 0.12 μL/min. The hydraulic resistance of the microchannel is calculated by multiplying the cross-sectional hydraulic resistance parameter *R*_cr_ (Eq. 5) with the microchannel length *L*. In our case the 20-turn serpentine microchannel has a total resistance of 0.12 μm^−3^, while in Zimmerman’s the 30 × 30 μm^2^ cross section of the 1-cm-long microchannel (Zimmermann et al. 2007) results in a hydraulic resistance of 0.35 μm^−3^ (around three times higher). Furthermore, comparing the average flow rate of our 20-turn diamond 50-μm MFS devices (0.96 μL/min) to the 15-μm characteristic dimension “symmetric-line”-type capillary devices of Zimmerman et al. (15_R1 has a flow rate of 0.22 μL/min (Zimmermann et al. 2007)), our devices have more than four times the flow rate, primarily due to the lower hydraulic resistance of the microchannel. The observed change in flow rate of the PCB-based capillary pumps can be attributed to the superhydrophilic properties of the O_2_ plasma treated FR-4 surface instead of the weakly hydrophilised Au surface (contact angle of 40°) in Zimmerman’s work (2007).

## Conclusions

The work presented here demonstrates a simple passive capillary pump that can be integrated into an affordable LoPCB platform. The microfluidic systems are based entirely on PCB manufacturing technology and will deliver low-cost, electronic-based disposable analytical platforms. We have demonstrated high-speed filling of LoPCB chips without requiring any external power source. Capillary pumps with an array of diamond shapes with particular geometrical parameters increase the induced capillary pressure and decrease the pillar hydraulic resistance. These designs, combined with the superhydrophilic properties of O_2_ irradiated FR-4 substrates, give average flow rates of around 8 μL/min. Increasing the channel length gives slower flow rates (1.43 μL/min ± 6% using a 10-turn serpentine microchannel). Significantly lower flow rates (an order of magnitude) were observed for even longer microchannels (0.58 μL/min ± 6% using a 20-turn serpentine microchannel). The variation in mean flow rate for the diamond micropillars with MFS 150 μm is below 7% for both, high and low flow rates, giving constant filling of a device. Capillary pumps without microchannel exhibit very high flow rates of around 138 μL/min. This performance is around 56 times higher than the average flow rates quoted by Madadi et al. (2014) with pumps fabricated from glass-PDMS due to both the 6.7 times higher micropillar height (decreasing the device hydraulic resistance) and the superhydrophilic properties of the O_2_-treated FR-4 substrate. The high and stable flow rates of our PCB-based capillary pumps could lead to functional, accurate and most importantly versatile microfluidic systems. Future directions include more complicated microfluidic networks, incorporating merging junctions, to give platforms for complicated assays.

## Electronic supplementary material

Below is the link to the electronic supplementary material.

Supplementary material 1 (MP4 6519 kb)
